# Khinchin Families, Set Constructions, Partitions and Exponentials

**DOI:** 10.1007/s00009-023-02579-9

**Published:** 2024-02-01

**Authors:** Alicia Cantón, José L. Fernández, Pablo Fernández, Víctor J. Maciá

**Affiliations:** 1 Departamento de Matemática e Informática Aplicadas a las Ingenierías Civil y Naval, ETSIN, Universidad Politécnica de Madrid, Avenida de la Memoria 4, Ciudad Universitaria, 28040 Madrid, Spain; 2 Departamento de Matemáticas, Facultad de Ciencias, Universidad Autónoma de Madrid, Ciudad Universitaria de Cantoblanco s/n, 28049 Madrid, Spain

**Keywords:** Khinchin families, Hayman-admissible functions, exponentials, set constructions, partitions, analytic combinatorics, asymptotic formulae, local central limit theorem, Primary: 30B10, Secondary: 05A16, 11P82, 60Fxx

## Abstract

In this paper, we give a simple criterion to verify that functions of the form $e^g$ are in the Hayman class when *g* is a power series with nonnegative coefficients. Thus, using the Hayman and Báez-Duarte formulas, we obtain asymptotics for the coefficients of generating functions that arise in many examples of set construction in analytic combinatorics. This new criterion greatly simplifies the one obtained previously by the authors.

## Introduction

The present paper is a follow-up to [3]. There, following the lead of Hayman [11], Rosenbloom [14] and Báez-Duarte [2], a basic theory of the so called Khinchin families is laid out. The notions of Gaussian and strongly Gaussian power series (which include power series in the Hayman class) are developed, and it is shown how the asymptotic formulas of Hayman and Báez-Duarte (see Theorem 4.10) provide a convenient way of handling the asymptotic of coefficients of strongly Gaussian power series.

In this context, a particularly interesting question consists of establishing the asymptotic behavior of the coefficients of a power series *f* that is written as $f= e^g$, where *g* is a power series with nonnegative coefficients.

For instance, in Combinatorics, the *set construction*, both labeled and unlabeled, is concerned with the combinatorial class of sets formed with objects drawn from a given combinatorial class. The generating function of the class of sets is of the form $f=e^g$, where *typically* *g* is a power series with nonnegative coefficients. The generating function of the Bell numbers and the partition function are examples of functions arising from the set construction. See Sect. 3 of the present paper, or consult the comprehensive treatise [6] of Flajolet and Sedgewick.

Hayman, in [11], deals with this question in the following particular cases: when *g* (not necessarily with nonnegative coefficients) already belongs to the Hayman class, and when *g* is a polynomial with nonnegative coefficients that obey certain divisibility properties. See the beginning of Sect. 5 for details.

In Theorem 4.1 of [3], a basic criterion is presented that can be used to establish conditions on the power series *g* with nonnegative coefficients which imply that $f=e^g$ is in the Hayman class and, therefore, is strongly Gaussian. This criterion, combined with the Hayman and Báez-Duarte asymptotic formulas, gives asymptotic results for the coefficients of a large and varied collection of power series.

In this paper, this criterion is greatly simplified and its applicability expanded. Theorems 5.5 and 5.7, the main results of the present paper, exhibit conditions on the (nonnegative) coefficients of a power series *g* which guarantee that $e^g$ is in the Hayman class. The criteria mentioned above are written directly in terms of the *growth* of the coefficients of *g*. Compare with Theorem 4.1 in [3], that is reproduced as Theorem G in this paper.

By appealing to Theorems 5.5 and 5.7, the verification that the generating functions of many combinatorial set constructions is in the Hayman class becomes immediate; asymptotic formulas for their coefficients follow simply from the Hayman and Báez-Duarte asymptotic formulas.

Although this paper is a follow-up to [3], we intend this presentation to be self-contained. The most salient and relevant features of the theory of Khinchin families are described within the present paper; nonetheless, we refer to [3] for a detailed treatment.

### Notation and Some Preliminaries

The symbol $a_n\sim b_n$ as $n\rightarrow \infty$ means that $a_n/b_n\rightarrow 1$ as $n\rightarrow \infty$, while $a_n\asymp b_n$ as $n\rightarrow \infty$ means that $1/C\le a_n/b_n<C$ for some $C>0$.

We use $\textbf{E}$, $\textbf{V}$ and $\textbf{P}$ to denote expectation, variance and probability generically.

For random variables *X* and *Y*, we write $X{\mathop {=}\limits ^{d}}Y$ to signify that *X* and *Y* have the same distribution.

If $(Z_n)_{n \ge 1}$ is a sequence of random variables and *Z* is another random variable, the notation $Z_n {\mathop {\longrightarrow }\limits ^\textrm{d}} Z$ as $n\rightarrow \infty$ means convergence in distribution, which by Levy’s theorem is equivalent to pointwise convergence of characteristic functions, i.e.,

$$\begin{aligned} \lim _{n \rightarrow \infty } \textbf{E}(e^{ \imath \theta Z_n}) =\textbf{E}(e^{ \imath \theta Z}), \quad \text{ for } \text{ every }\ \theta \in {\mathbb {R}}. \end{aligned}$$

For sums of power of divisors of integers, we use the following notations. For any integer $c \ge 0$, we denote with $\sigma _c(m)$ the sum of the *c*th powers of the divisors of *m*:

$$\begin{aligned} \sigma _c(m)=\sum _{j\mid m} j^c, \quad \text{ for }\ m \ge 1. \end{aligned}$$

Moreover, $\sigma _c^{\textrm{odd}}(m)$ denotes the analogous sum but restricted to the odd divisors of *m*:

$$\begin{aligned} \sigma _c^{\textrm{odd}}(m)=\sum _{j\mid m,\, j \,\textrm{odd}} j^c,\quad \text{ for }\ m \ge 1. \end{aligned}$$

### Plan of the Paper

Section 2 covers the basic background material on Khinchin families, the framework of the present paper, while Sect. 4 focuses on Gaussian and strongly Gaussian Khinchin families and the Hayman class. The reference [3] covers Khinchin families at length.

Section 3 describes the fundamental set constructions from the point of view of Khinchin families and the exponential function, which furnishes the basic context of application of the results of this paper.

The basic criteria for verifying that the exponential of a power series of nonnegative coefficients is in the Hayman class are the main results of Sect. 5.

Finally, in Sect. 6, we briefly recall the procedure to obtain asymptotic formulas of coefficients of strongly Gaussian power series.

## Khinchin Families

We denote by ${\mathcal {K}}$ the class of *nonconstant* power series $f(z)=\sum _{n=0}^\infty a_n z^n$ with positive radius of convergence, which have *nonnegative* Taylor coefficients, $a_n \ge 0$, for each $n \ge 0$, and such that $a_0>0$.

The *Khinchin family* of such a power series $f \in {\mathcal {K}}$ with radius of convergence $R>0$ is the family of random variables $(X_t)_{t \in [0,R)}$ with values in $\{0, 1, \ldots \}$ and with mass functions given by

$$\begin{aligned} \textbf{P}(X_t=n)=\frac{a_n t^n}{f(t)}, \quad \text{ for } \text{ each }\ n \ge 0 \ \text{ and }\ t\in (0,R). \end{aligned}$$

The variable $X_0$ of the family is defined as $X_0\equiv 0$. Notice that $f(t)>0$ for each $t \in [0,R)$.

Any Khinchin family is continuous in distribution in [0, *R*), in the sense that if a sequence $(s_n)_{n \ge 1} \subset [0,R)$ converges to $s_0\in [0,R)$, then $X_{s_n}{\mathop {\longrightarrow }\limits ^\textrm{d}} X_{s_0}$, as $n \rightarrow \infty$ (see, for instance, [5]). Observe that no hypothesis upon joint distribution of the variables $X_t$ is considered; $(X_t)_{t \in [0,R)}$ is a family, not a process.

### Basic Properties

For the basic theory of Khinchin families (results, proofs, examples and applications), we refer the reader to [3]. Here we describe the specific aspects of the theory to be used in the present paper.

#### Mean and Variance Functions

For the mean and variance of $X_t$ we reserve the notation $m(t)=\textbf{E}(X_t)$ and $\sigma ^2(t)=\textbf{V}(X_t)$, for $t \in [0,R)$. In terms of *f*, the mean and the variance of $X_t$ may be written as

$$\begin{aligned} m(t)=\frac{t f^\prime (t)}{f(t)}, \qquad \sigma ^2(t)=t m^\prime (t), \quad \text{ for }\ t \in [0,R). \end{aligned}$$

For each $t \in (0,R)$, the variable $X_t$ is not a constant, and so $\sigma ^2(t)>0$. Consequently, *m*(*t*) is strictly increasing in [0, *R*), though, in general, $\sigma (t)$ is not increasing. We denote

$$\begin{aligned} M_f=\lim _{t \uparrow R} m(t). \end{aligned}$$

Whenever $M_f=+\infty$, for each integer $n \ge 0$, we use $t_n$ to denote the unique $t_n\in [0,R)$, such that $m(t_n)=n$.

#### Normalization and Characteristic Functions

For each $t \in (0,R)$, the normalization of $X_t$ is

$$\begin{aligned} \breve{X}_t\triangleq \frac{X_t-m(t)}{\sigma (t)}\,\cdot \end{aligned}$$

The characteristic function of the variable $X_t$ may be written in terms of the power series *f* itself as

$$\begin{aligned} \textbf{E}(e^{\imath \theta X_t})=\frac{f(te^{\imath \theta })}{f(t)}, \quad \text{ for }\ t\in (0,R)\ \text{ and }\ \theta \in {\mathbb {R}}, \end{aligned}$$

while for its normalized version $\breve{X}_t$ we have that

$$\begin{aligned} \textbf{E}(e^{\imath \theta \breve{X}_t})=\textbf{E}(e^{\imath \theta X_t/ \sigma (t)}) \,e^{-\imath \theta m(t)/\sigma (t)}, \quad \text{ for }\ t\in (0,R)\ \text{ and }\ \theta \in {\mathbb {R}}, \end{aligned}$$

and so,

$$\begin{aligned} |\textbf{E}(e^{\imath \theta \breve{X}_t})|=|\textbf{E}(e^{\imath \theta X_t/\sigma (t)})|, \quad \text{ for }\ t\in (0,R) \ \text{ and }\ \theta \in {\mathbb {R}}. \end{aligned}$$

#### Fulcrum *F* of *f*

The holomorphic function *f* does not vanish on the real interval [0, *R*), and so, it does not vanish in a simply connected region containing that interval. We may consider $\ln f$, a holomorphic branch of the logarithm of *f* which is real on [0, *R*), and the function *F*, which we shall call the *fulcrum of* *f*, defined and holomorphic in a region containing $(-\infty , \ln R)$, and which is given by

$$\begin{aligned} F(z)=\ln f(e^z). \end{aligned}$$

If *f* does not vanish anywhere in the disk ${\mathbb {D}}(0,R)$, then the fulcrum *F* of *f* is defined in the whole half plane $\{z\in {\mathbb {C}}: \Re z< \ln R\}$. In this paper, this situation of *f* nonvanishing in ${\mathbb {D}}(0,R)$ is the most interesting. In this case $f(z)=e^{g(z)}$, where *g* is a function holomorphic in ${\mathbb {D}}(0,R)$ and $g(0)\in {\mathbb {R}}$, and the fulcrum *F* of *f* may be written as

$$\begin{aligned} F(z)=g(e^z), \quad \text{ for }\ z\ \text{ such } \text{ that }\ \Re z<\ln R. \end{aligned}$$

The mean and variance function of *f* may be expressed in terms of its fulcrum *F* as

$$\begin{aligned} m(t)=F^\prime (s)\quad \text{ and } \quad \sigma ^2(t) =F^{\prime \prime }(s), \quad \text{ for }\ s <\ln R\ \text{ and }\ t=e^s. \end{aligned}$$

### Hayman’s Identity

For a power series $f(z)=\sum _{n=0}^\infty a_n z^n$ in ${\mathcal {K}}$, Cauchy’s formula for the coefficient $a_n$ may be written in terms of the characteristic function of its Khinchin family $(X_t)_{t \in [0,R)}$ as

$$\begin{aligned} a_n=\frac{f(t)}{2\pi t^n }\int _{|\theta |<\pi } \textbf{E}(e^{\imath \theta X_t})\, e^{-\imath \theta n} \, \mathrm{{d}} \theta , \quad \text{ for } \text{ each }\ t \in (0,R)\ \text{ and }\ n\ge 1. \end{aligned}$$

In terms of the characteristic function of the normalized variable $\breve{X}_t$, Cauchy’s formula becomes

$$\begin{aligned} a_n= & {} \frac{f(t)}{2\pi \,t^n \,\sigma (t)}\int \nolimits _{|\theta | <\pi \sigma (t)} \textbf{E}(e^{\imath \theta \breve{X}_t}) \, e^{-\imath \theta (n-m(t))/\sigma (t)} \, \mathrm{{d}} \theta \\{} & {} \qquad \qquad \quad \qquad \qquad \qquad \text{ for }\ t \in (0,R)\ \text{ and }\ n \ge 1. \end{aligned}$$

If $M_f=\infty$, we may take for each $n\ge 1$ the (unique) radius $t_n \in (0,R)$ so that $m(t_n)=n$, to write

2.1 $$\begin{aligned} a_n=\frac{f(t_n)}{2\pi \,t_n^n \,\sigma (t_n)} \int _{|\theta |<\pi \sigma (t_n)} \textbf{E}(e^{\imath \theta \breve{X}_{t_n}}) \, \mathrm{{d}} \theta , \quad \text{ for } \text{ each }\ n \ge 1, \end{aligned}$$

which we call *Hayman’s identity*.

This identity (2.1), which is just Cauchy’s formula with an appropriate choice of radius $t_n$, neatly encapsulates, in fact, the saddle point method.

### Basic Khinchin Families

The most basic collections of probability distributions in $\{0,1, \ldots \}$, i.e., Bernoulli and binomial, geometric and negative binomial and Poisson, are (the most basic) Khinchin families. A quick review follows; see more details, for example, in Section 2.1.6 of [3].

- The Khinchin family of the function $f(z)=1+z$ consists of the *Bernoulli variables*.
  In this case, $R=\infty$, and the mean and variance functions are $m(t)=t/(1+t)$ and $\sigma ^2(t)=t/(1+t)^2$. For each $t >0$, the random variable $X_t$ is a *Bernoulli* variable with parameter $p=t/(1+t)$.
- Let $f(z)=(1+z)^N$, with integer $N \ge 1$. This is the *binomial case*.
  In this case, $R=\infty$, and the mean and variance functions are $m(t)=Nt/(1+t)$ and $\sigma ^2(t)=Nt/(1+t)^2$. For each $t >0$, the random variable $X_t$ is a *binomial* variable with parameters *N* and $p=t/(1+t)$.
- The function $f(z)=1/(1-z)$ corresponds to the *geometric case*.
  In this case $R=1$, and the mean and variance functions are $m(t)=t/(1-t)$ and $\sigma ^2(t)=t/(1-t)^2$. For each $t\in (0,1)$, the random variable $X_t$ is a *geometric* variable (number of failures until first success) of parameter $1-t$, that is, $\textbf{P}(X_t=k)=t^{k} (1-t)$ for $k \ge 0$.
- Let $f(z)=1/(1-z)^N$, with integer $N \ge 1$; the *negative binomial* case.
  In this case $R=1$, and the mean and variance functions are $m(t)=N t/(1-t)$ and $\sigma ^2(t)=N t/(1-t)^2$. For each $t\in (0,1)$, the random variable $X_t$ is a *negative binomial* variable of parameters $N \ge 1$ and $p=1-t$.
- The Khinchin family of the exponential function $f(z)=e^z$ consists of the Poisson variables: the *Poisson case*.
  In this case $R=\infty$, and the mean and variance functions are $m(t)=t$ and $\sigma ^2(t)=t$. For each $t>0$, the random variable $X_t$ in its Khinchin family is a *Poisson* variable with parameter *t*.

### A Couple of Power Series Comparisons

In the proofs of Theorems 5.5 and 5.7, the main results of this paper, we will resort to the following asymptotics for a couple of series.

#### Proposition 2.1

For $$\beta >0$$,

$$\begin{aligned} \sum _{n=1}^\infty n^{\beta -1}\, t^n \sim \Gamma (\beta ) \,\frac{1}{(1-t)^{\beta }}, \quad \text{ as }\ t\uparrow 1. \end{aligned}$$

#### Proof

The binomial expansion gives that

$$\begin{aligned} \frac{1}{(1-z)^\beta }=\sum _{n=0}^\infty \frac{\Gamma (n+\beta )}{\Gamma (\beta )\, n!} \, z^n,\quad \text{ for }\ |z|<1. \end{aligned}$$

And Stirling’s formula gives, for $\beta >0$, that

2.2 $$\begin{aligned} \frac{\Gamma (n+\beta )}{\Gamma (\beta )\, n!} \sim \frac{n^{\beta -1}}{\Gamma (\beta )}, \quad \text{ as }\ n \rightarrow \infty . \end{aligned}$$

Thus,

$$\begin{aligned} \sum _{n=1}^\infty n^{\beta -1} \, t^n \sim \Gamma (\beta ) \, \frac{1}{(1-t)^{\beta }}, \quad \text{ as }\ t\uparrow 1. \end{aligned}$$

$$\square $$

#### Remark 2.2

(Moments of geometric random variables) The power series comparison of Proposition 2.1 translates into a comparison of moments of the Khinchin family $(X_t)_{t \in [0,1)}$ of $1/(1-z)$, i.e., of geometric variables. Namely, the following: for $\beta >0$,

$$\begin{aligned} \textbf{E}(X_t^\beta )\sim \Gamma (\beta +1)\,\textbf{E}(X_t)^\beta , \quad \text{ as }\ t \uparrow 1. \end{aligned}$$

For $\beta \in (-1,0)$, the same result holds for the variables $\tilde{X}_t$, the $X_t$ *conditioned at being positive*, given by $\textbf{P}(\tilde{X}_t=k)=t^{k{-}1} (1-t)$ for $k \ge 1$ and $t \in (0,1)$.

#### Proposition 2.3

For $\beta \ge 0$, we have that

$$\begin{aligned} \sum _{n=0}^\infty n^\beta \,\frac{t^n}{n!}\sim t^\beta e^t \quad \text {and}\quad \sum _{n=1}^\infty \frac{1}{n^\beta } \,\frac{t^n}{n!}\sim \frac{e^t}{t^\beta } \quad \text{ as }\ t \rightarrow \infty . \end{aligned}$$

#### Proof

Upon derivation of $e^t=\sum _{n=0}^\infty t^n/n!$, we deduce that for any integer $k\ge 1$,

$$\begin{aligned} \sum _{n=0}^\infty \frac{n(n-1)\cdots (n-k+1)}{n!}\, t^n =t^k \, e^t,\quad \text {for}\ t\in {\mathbb {R}}. \end{aligned}$$

From there, it follows that

$$\begin{aligned} \sum _{n=0}^\infty \frac{n^k}{n!}\, t^n \sim t^k\, e^t, \quad \text {as}\ t\rightarrow \infty . \end{aligned}$$

Convexity and Jensen’s inequality imply that, for $\beta >0$,

$$\begin{aligned} \sum _{n=0}^\infty \frac{n^\beta }{n!}\, t^n \sim t^\beta \, e^t, \quad \text {as}\ t\rightarrow \infty . \end{aligned}$$

For negative exponents, we refer to [12] and to Remark 2.4 below. $\square$

#### Remark 2.4

(Moments of Poisson random variables) Proposition 2.3 is actually a statement about positive moments of the Poisson variables $X_t$, and also about negative moments of the conditioned Poisson variables $\tilde{X}_t$, as the mean *t* tends to $\infty$. The conditioned Poisson variables $\tilde{X}_t$ are given by

$$\begin{aligned} \textbf{P}(\tilde{X}_t=k)=\frac{e^{-t}}{1-e^{-t}} \,\frac{t^k}{k!}, \quad \text{ for }\ k \ge 1\ \text{ and }\ t >0. \end{aligned}$$

This moments estimation is the following: if $(X_t)_{t \in [0,1)}$ is the Khinchin family of $e^z$, then for any $\beta \ge 0$,

$$\begin{aligned} \textbf{E}(X_t^\beta )\sim t^\beta , \quad \text{ as }\ t \rightarrow \infty , \end{aligned}$$

and for any $\beta <0$,

$$\begin{aligned} \textbf{E}\big (\tilde{X}_t^\beta \big )\sim t^\beta , \quad \text{ as }\ t \rightarrow \infty . \end{aligned}$$

See also [12] for precise asymptotic *expansions* of the negative moments of Poisson variables.

## Set Construction and Khinchin Families

The *set construction of combinatorial classes* fits nicely within the framework of Khinchin families. As a general reference for combinatorial classes and operations with them, we strongly suggest Chapter II in Flajolet–Sedgewick [6]. See also [1], where the constructions below are *presented* as *decomposable structures*.

We shall abbreviate ‘exponential generating function’ by egf, and ‘ordinary generating function’ by ogf.

Most of the (ordinary or exponential) generating functions of sets of combinatorial classes are exponentials of power series with nonnegative coefficients, *the object of interest* of this paper.

We will verify by means of Theorems 5.5 and 5.7 that most of the set constructions give rise to generating functions which are in the Hayman class, see Sect. 4.3, so that there are asymptotic formulas for their coefficients given by the Hayman and Báez-Duarte asymptotic formulas of Sect. 4.2.1.

### Labeled Combinatorial Classes and Sets

If $g(z)=\sum _{n=1}^\infty (b_n/n!) \,z^n$ is the exponential generating function (egf) of a labeled combinatorial class ${\mathcal {G}}$ (no object of size 0), then $f(z)=e^{g(z)}=\sum _{n=0}^{\infty }(a_n/n!) z^n$ is the egf of the labeled class of sets formed with the objects of ${\mathcal {G}}$, termed *assemblies* in [1].

Next, we consider first sets of the basic classes: sets, lists and cycles, and then sets of rooted trees and of functions.

#### Sets of Sets

Let ${\mathcal {G}}$ be the labeled class of nonempty sets: $b_n=1$, for each $n \ge 1$, and $b_0=0$. Its egf is $g(z)=e^z-1$. Then

$$\begin{aligned} f(z)=\exp (e^z-1)=\sum _{n=0}^\infty \frac{B_n}{n!}\, z^n \end{aligned}$$

is the egf of sets of sets, or, equivalently, of partitions of sets, see [6], p. 107. Here, $B_n$ is the *n*th Bell number, which counts the number of partitions of the set $\{1, \ldots , n\}$. In this case $R=\infty$, and the mean and variance function are $m(t)=t e^t$ and $\sigma ^2(t)=t(t+1)e^t$.

The characteristic function of the Khinchin family of *f* is given by

$$\begin{aligned} \textbf{E}(e^{\imath \theta X_t})=\exp (e^{t e^{\imath \theta }}-e^t ), \quad \text{ for }\ \theta \in {\mathbb {R}}\ \text{ and }\ t >0, \end{aligned}$$

and thus, for $\theta \in {\mathbb {R}}$ and $t >0$,

3.1 $$\begin{aligned} \textbf{E}(e^{\imath \theta \breve{X}_t})=\exp \big (e^{t e^{\imath \theta {e^{-t/2}}/{\sqrt{t(t+1)}}}}-e^t-\imath \theta \sqrt{t/(t+1)}e^{t/2}\big ). \end{aligned}$$

$\bullet$ The class ${\mathcal {G}}$ of pointed sets (i.e., sets with one marked element) has egf $g(z)=ze^z$. The class of sets of pointed sets is isomorphic to the class of idempotent maps; its egf *f* is given by $f(z)=\exp (ze^z)$. See [6], p. 131.

#### Sets of Lists

For the labeled class ${\mathcal {G}}$ of (nonempty) lists, the function *g* is just $g(z)=z/(1-z)$. And the function $f(z)=\exp (z/(1-z))$ is the egf of the sets of lists, the so called fragmented permutations. See [6], p. 125.

#### Sets of Cycles

The labeled class ${\mathcal {G}}$ of (nonempty) cycles has $g(z)=\ln (1/(1-z))$ as egf. The function $f(z)=\exp (g(z))={1}/(1-z)$ is the egf of the sets of cycles, or, in other terms, of the permutations.

$\bullet$ The length of the cycles could be restricted. Thus, for integer $k \ge 1$,

$$\begin{aligned} \exp \Big (\sum _{n\le k} z^n/n\Big )\quad \text {or}\quad \exp \Big (\sum _{n\ge k} z^n/n\Big ) \end{aligned}$$

are the egfs of permutations such that all the cycles in their cycle decomposition have length at most *k*, or at least *k*, respectively. We may also consider, for $k \ge 1$,

$$\begin{aligned} \exp \Big (\sum _{d\ge 1,\, d\mid k} z^d/d\Big ), \end{aligned}$$

which is the egf of the permutations $\sigma$ such that $\sigma ^k$ is the identity.

#### Sets of Trees and Sets of Functions

$\bullet$ *Sets of trees* (*forests*). The class ${\mathcal {G}}$ of rooted (labeled) trees has egf $g(z)=\sum _{n=1}^\infty (n^{n-1}/n!) \,z^n$. See [6], Section II.5.1; this is Cayley’s theorem. The class of forests (sets) of rooted (labeled) trees has then egf $f=e^g$.

Cayley’s theorem also shows that the egf of the class of unrooted (labeled) trees is $g(z)=\sum _{n=1}^\infty (n^{n-2}/n!)\, z^n$.

$\bullet$ *Sets of functions*. The class ${\mathcal {G}}$ of functions has egf $g(z)=\sum _{n=1}^\infty (n^n/n!) z^n$. See [6], Section II.5.2. The class of sets of functions has then egf $f=e^g$.

### Unlabeled Combinatorial Classes and Sets

We split the discussion into multisets and (proper) sets. See Section I.2.2 in [6] and [1] as general references for the set constructions of unlabeled combinatorial classes.

#### Multisets of Unlabeled Combinatorial Classes

If $C(z)=\sum _{n=1}^\infty c_n z^n$ is the ordinary generating function (ogf) of a combinatorial (unlabeled) class ${\mathcal {G}}$ (no object of size 0), then

$$\begin{aligned} f(z)=\prod _{j=1}^\infty \frac{1}{(1-z^j)^{c_j}} =\exp \Big (\sum _{n=1}^\infty C(z^n)/n\Big ) \end{aligned}$$

is the ogf of the *class of sets formed with the objects of* ${\mathcal {G}}$, termed *multisets* in [1].

We may write $f(z)=e^{g(z)}$, where *g* is the power series

$$\begin{aligned} g(z)=\sum _{j,k\ge 1} c_j\,\frac{z^{kj}}{k} =\sum _{m=1}^\infty \Big (\sum _{j \mid m}j c_j\Big ) \frac{z^m}{m}\triangleq \sum _{m=1}^\infty b_m\, z^m. \end{aligned}$$

Observe that the power series *g* has nonnegative coefficients:

$$\begin{aligned} b_m=\frac{1}{m}\sum _{j \mid m}j c_j, \quad \text{ for }\ m\ge 1. \end{aligned}$$

$\bullet$ The ogf of partitions, *the partition function*, given by

$$\begin{aligned} P(z)=\prod _{j=1}^\infty \frac{1}{1-z^j}=\sum _{n=0}^\infty p(n) \, z^n, \quad \text{ for }\ z \in {\mathbb {D}}, \end{aligned}$$

is in ${\mathcal {K}}$. Here *p*(*n*) is the number of partitions of the integer $n \ge 1$, and $p(0)=1$.

The mean and variance functions of its Khinchin family are not so direct. In this instance, *C* is $C(z)=z/(1-z)$, as $c_n=1$ for $n \ge 1$ (one object of weight *n*, for each $n \ge 1$). And thus *g* has coefficients $b_m=\sigma _1(m)/m$, where $\sigma _1(m)$ is the sum of the divisors of the integer *m*.

For general $C(z)=\sum _{n=1}^\infty c_n z^n$ as above, the corresponding *f* is the ogf of the colored partitions; $c_j$ different colors for part *j*.

#### Sets of Unlabeled Combinatorial Classes

If again we write $C(z)=\sum _{n=1}^\infty c_n z^n$ for the ordinary generating function (ogf) of a combinatorial (unlabeled) class ${\mathcal {G}}$ (no object of size 0), then

$$\begin{aligned} f(z)=\prod _{j=1}^\infty (1+z^j)^{c_j}=\exp \Big (\sum _{n=1}^\infty (-1)^{n+1}C(z^n)/n\Big ), \end{aligned}$$

is the ogf of the class of (proper) sets formed with the objects of ${\mathcal {G}}$, termed *selections* in [1].

We may write $f(z)=e^{g(z)}$, where *g* is the power series

$$\begin{aligned} g(z)=\sum _{j,k\ge 1} c_j\,\frac{z^{kj}\,(-1)^{k+1}}{k} =\sum _{m=1}^\infty \Big (\sum _{jk=m}j c_j\,(-1)^{k+1}\Big ) \frac{z^m}{m}\,\cdot \end{aligned}$$

In general, the power series *g* could have negative coefficients; this is the case, for instance, for $f(z)=(1+z)^5 (1+z^2)$.

But for the sequence of coefficients of *C* given by $c_j=j^{c-1}, j \ge 1$, where *c* is an integer $c\ge 1$, *the coefficients of* *g* *are nonnegative*; in fact, the *m*th coefficient of *g* is

$$\begin{aligned} \frac{1}{m} \,\sigma _c^{\textrm{odd}}(m) \,\omega (m), \end{aligned}$$

where $\omega (m)$ is given by

$$\begin{aligned} \omega (m)=\frac{2^c-2}{2^c-1}\, 2^{\chi (m)c} +\frac{1}{2^c-1}, \quad \text{ for }\ m \ge 1, \end{aligned}$$

$\chi (m)$ is the highest integer exponent so that $2^{\chi (m)}\mid m$, and $\sigma _c^{\textrm{odd}}(m)$ is the sum of the *c*th powers of the odd divisors of *m*. This is so because of the identity

3.2 $$\begin{aligned} \sum _{jk=m}j^c (-1)^{k+1}=\Big (\sum _{j\mid m,\, j\, \textrm{odd}} j^c\Big ) \, \omega (m), \quad \text{ for }\ m \ge 1. \end{aligned}$$

To verify (3.2), observe first that as functions of *m*, both summations in (3.2) are multiplicative. Write *m* as $m=2^{\chi (m)} s$, with *s* odd, and observe that for $m=2^r$, with $r \ge 1$, the summation on the left is $\omega (m)$ and the summation on the right is 1, while for $m=s$ odd, the two sums coincide and $\omega (s)=1$.

We may also write

$$\begin{aligned} \sum _{jk=m}j^c (-1)^{k+1}=\sigma _c(m)-2 \sigma _c(m/2), \quad \text{ for }\ m \ge 1, \end{aligned}$$

with the understanding that $\sigma _c(m/2)=0$ if *m* is odd. For the identity (3.2) and a variety of relations among a number of diverse sums on divisors, we refer to [8].

More generally, if the coefficient $c_j$ of *C* is given by $c_j=R(j)$, for $j \ge 1$, where *R*(*z*) is a polynomial with nonnegative integer coefficients, then the coefficients of *g* are nonnegative.

$\bullet$ The ogf of *partitions with distinct parts*, given by

$$\begin{aligned} Q(z)=\prod _{j=1}^\infty (1+z^j)=\sum _{n=0}^\infty q(n)\, z^n, \quad \text{ for }\ z \in {\mathbb {D}}, \end{aligned}$$

is in ${\mathcal {K}}$. Here *q*(*n*) is the number of partitions into distinct parts of the integer $n \ge 1$.

In this particular instance, the power series *C* is $C(z)=z/(1-z)$, as $c_n=1$, for $n \ge 1$, and the power series *g* is

$$\begin{aligned} g(z)=\sum _{m=1}^\infty \frac{\sigma _1(m)-2\sigma _1(m/2)}{m}\, z^m. \end{aligned}$$

## Gaussian Khinchin Families and Hayman Class

### Definition 4.1

A power series $f \in {\mathcal {K}}$ and its Khinchin family $(X_t)_{t \in [0,R)}$ are termed *Gaussian* if the normalized sequence $(\breve{X}_t)$ converges in distribution, as $t\uparrow R$, to the standard normal or, equivalently, if

$$\begin{aligned} \lim _{t \uparrow R} \textbf{E}(e^{\imath \theta \breve{X}_t})=e^{-\theta ^2/2}, \quad \text{ for } \text{ each }\ \theta \in {\mathbb {R}}. \end{aligned}$$

Among the basic Khinchin families considered in Sect. 2.3, only the family associated to the exponential $e^z$ is Gaussian.

For the functions $f(z)=1+z$ (Bernoulli case) or $f(z)=(1+z)^N$ (binomial case), the corresponding $(\breve{X}_t)$ converges in distribution, as $t\rightarrow \infty$, to the constant 0. In fact, for any polynomial in ${\mathcal {K}}$, the corresponding $(\breve{X}_t)$ converges in distribution, as $t\rightarrow \infty$, towards the constant 0.

For the function $f(z)=1/(1-z)$ (geometric case), $(\breve{X}_t)$ converges in distribution, as $t\uparrow 1$, towards a variable *Z*, where $Z+1$ is an exponential variable of parameter 1. For $f(z)=1/(1-z)^N$, with integer $N\ge 1$ (negative binomial case), it can be verified that $(\breve{X}_t)$ converges in distribution, as $t\uparrow 1$, towards a variable $Z_N$, where $Z_N+\sqrt{N}$ follows a Gamma distribution with shape parameter *N* and rate parameter $\sqrt{N}$ (or scale parameter $1/\sqrt{N}$).

Thus, in all the cases considered above, $(\breve{X}_t)$ converges in distribution as $t \uparrow R$, but only for the exponential $e^z$ the limit is the (standard) normal distribution. See details in Section 3.1 of [3].

### Gaussianity of Exponentials

The following Theorem A is the basic criterion for gaussianity in terms of the fulcrum *F* of *f* (of Sect. 2.1.3); it originates in Hayman’s [11], but we refer to Theorem 3.2 in [3] for a proof.

#### Theorem A

If $f\in {\mathcal {K}}$ has radius of convergence $R>0$ and vanishes nowhere in ${\mathbb {D}}(0,R)$, and if for the fulcrum *F* of *f* one has

4.1 $$\begin{aligned} \lim _{s \uparrow \ln R} \frac{\sup _{\phi \in {\mathbb {R}}} \big |F^{\prime \prime \prime }(s+i\phi )\big |}{F^{\prime \prime }(s)^{3/2}}=0, \end{aligned}$$

then *f* is Gaussian.

Notice that Theorem A applies whenever $f \in {\mathcal {K}}$ is of the form $f=e^g$ for some power series *g*, that may have negative Taylor coefficients. As registered in the following Theorem B, if $f=e^g$ and *g* has nonnegative coefficients, a simpler condition on *g* implies the gaussianity of $f=e^g$.

#### Theorem B

Let $f\in {\mathcal {K}}$ be such that $f=e^g$, where *g* has radius of convergence $R>0$ and nonnegative coefficients. If *g* is a polynomial of degree 1 or *g* satisfies

4.2 $$\begin{aligned} \lim _{t \uparrow R} \frac{g^{\prime \prime \prime }(t)}{{g^{\prime \prime }(t)}^{3/2}}=0, \end{aligned}$$

then *f* is Gaussian.

For a proof, see Theorem 3.3 in [3].

$\bullet$ For the exponential function $f(z)=e^z$, we have that its fulcrum is $F(z)=e^z$, and its gaussianity follows readily from Theorem A.

$\bullet$ More generally, if $B(z)=\sum _{j=0}^N b_j \, z^j$ is a polynomial of degree *N* such that $e^{B}\in {\mathcal {K}}$, then $e^{B}$ is Gaussian. For, in this case, $F(z)=B(e^z)$, and for $z=s+\imath \phi$ we have that

$$\begin{aligned} |F^{\prime \prime \prime }(z)|=\Big |\sum _{j=0}^N b_j \,j^3 e^{jz}\Big | \le \sum _{j=0}^N |b_j| \,j^3 e^{js}=O(e^{Ns}), \end{aligned}$$

while

4.3 $$\begin{aligned} F^{\prime \prime }(s)=\sum _{j=0}^N b_j \,j^2 e^{js}\sim b_N \,N^2 e^{Ns}\, \quad \text{ as }\ s \uparrow \infty , \end{aligned}$$

and gaussianity follows from Theorem A. Observe that (4.3) implies that $b_N$ is real and $b_N >0$; besides, since $e^{B} \in {\mathcal {K}}$, the coefficients of the polynomial *B* must be real numbers.

$\bullet$ For $f(z)=\exp (e^z-1)$, the egf of the *class of sets of sets*, and for $f(z)=\exp (z/(1-z))$, the egf of the *class of sets of lists*, Theorem A (or Theorem B) gives readily that both are Gaussian. Similarly, the function $f(z)=\exp (z e^z)$, the egf of the *class of sets of pointed sets*, is Gaussian.

$\bullet$ Again directly from Theorem A or Theorem B, we see that the functions $f(z)=\exp (1/(1-z)^\gamma )$, with $\gamma >0$, are all Gaussian. The functions

4.4 $$\begin{aligned} f(z)=\exp \Big (\sum _{n=1}^\infty n^\alpha z^n\Big ), \quad \text{ for }\ |z|<1, \end{aligned}$$

with $\alpha >-1$ are also Gaussian. Indeed, the fulcrum *F* of such an *f* is

$$\begin{aligned} F(z)=\sum _{n=1}^\infty n ^\alpha e^{n z}, \quad \text{ for }\ \Re z<0. \end{aligned}$$

Then, for $s<0$ and $r=e^s$, we have that

$$\begin{aligned} \sup _{\phi \in {\mathbb {R}}}|F^{\prime \prime \prime }(s+\imath \phi )| =\sum _{n=1}^\infty n^{\alpha +3} \,r^n,\quad \text{ while } \quad |F^{\prime \prime }(s)|=\sum _{n=1}^\infty n^{\alpha +2} \,r^n. \end{aligned}$$

By appealing to Proposition 2.1, we deduce that condition (4.1) is satisfied. Observe that $R=1$, in this case.

$\bullet$ In the same vein, the egf of *sets of functions* $f(z)=\exp \big (\sum _{n=1}^{\infty } ({n^n}/{n!}) z^n\big )$, with $R=1/e$, is also Gaussian. Similarly, and more generally,

$$\begin{aligned} f(z)=\exp \Big (\sum _{n=1}^\infty \frac{n^{n-\delta }}{n!} \,z^n\Big ) \end{aligned}$$

is Gaussian, for $0\le \delta <1/2$. To see this, observe (Stirling’s formula) that

$$\begin{aligned} \frac{n^{n-\delta }}{n!}\asymp \frac{e^n}{n^{\delta +1/2}}, \quad \text{ as }\ n \rightarrow \infty . \end{aligned}$$

and argue as with (4.4), taking there $\alpha =-\delta -1/2$.

$\bullet$ The *partition function* *P* *and the* ogf $\,Q$ *of partitions into distinct parts* are seen to be Gaussian as a consequence of Theorem A; but see Sect. 5 for the stronger statement that *P* and *Q* are both in the Hayman class.

Now we turn our attention to a couple of *non-examples*.

$\bullet$ The egf $f(z)=\exp (\ln (1/(1-z)))$ of the *class of sets of cycles*, which is simply $f(z)=1/(1-z)$, the egf of permutations, is *not Gaussian*, as we have mentioned at the beginning of this section. The condition of Theorem A is, of course, not satisfied; in fact, for the corresponding fulcrum $F(z)=-\ln (1-e^z)$, for $\Re z <0$, it holds that

$$\begin{aligned} \lim _{s \uparrow 0} \frac{\sup _{\phi \in {\mathbb {R}}} |F^{\prime \prime \prime } (s+i\phi )|}{F^{\prime \prime }(s)^{3/2}}=2. \end{aligned}$$

Analogously, the class of sets of cycles of length at least *k* is not Gaussian, for any $k \ge 1$.

$\bullet$ The egf $f(z)=\exp \big (\sum _{n=1}^\infty ({n^{n-1}}/{n!}) z^n\big )$ of forests of rooted trees is *not Gaussian*. In fact, we are going to check that the characteristic functions of its normalized Khinchin family converge in distribution to the constant 1.

The power series in the expression of *f* has radius of convergence $R=1/e$, and

$$\begin{aligned} \lim _{t\uparrow 1/e} f(t)=\exp \Big (\sum _{n=1}^\infty \frac{n^{n-1}}{n!\, e^n}\Big )<+\infty , \end{aligned}$$

since

$$\begin{aligned} \frac{n^{n-1}}{n!\, e^n}\asymp \frac{1}{n^{3/2}} \quad \text {as}\ n\rightarrow \infty . \end{aligned}$$

In particular, the function *f* extends to be continuous in $\textrm{cl}({\mathbb {D}}(0,1/e))$.

Now

$$\begin{aligned} m(t)=\sum _{n=1}^\infty \frac{n^{n}}{n!} \,t^n, \quad \text{ for }\ t \in (0,1/e), \end{aligned}$$

and Proposition 2.1 gives that

$$\begin{aligned} m(t/e)=\sum _{n=1}^\infty \frac{n^{n}}{n!\,e^n} \,t^n \asymp \frac{1}{(1-t)^{1/2}}, \quad \text{ as }\ t \uparrow 1. \end{aligned}$$

Also

$$\begin{aligned} \sigma ^2(t)=\sum _{n=1}^\infty \frac{n^{n+1}}{n!} \,t^n, \quad \text{ for }\ t \in (0,1/e), \end{aligned}$$

and Proposition 2.1 gives that

$$\begin{aligned} \sigma ^2(t/e)=\sum _{n=1}^\infty \frac{n^{n+1}}{n!\,e^n} \,t^n\asymp \frac{1}{(1-t)^{3/2}}, \quad \text{ as }\ t \uparrow 1. \end{aligned}$$

Thus

$$\begin{aligned} \frac{\sigma (t/e)}{m(t/e)}\asymp \frac{1}{(1-t)^{1/4}}, \quad \text{ as }\ t \uparrow 1, \end{aligned}$$

and so, in particular,

4.5 $$\begin{aligned} \lim _{t \uparrow 1/e} \frac{m(t)}{\sigma (t)}=0. \end{aligned}$$

Now for $\theta \in {\mathbb {R}}$ we may write

$$\begin{aligned} \textbf{E}(e^{\imath \theta \breve{X}_t}) =\frac{f(t e^{\imath \theta /\sigma (t)})}{f(t)} \, e^{-\imath \theta m(t)/\sigma (t)}. \end{aligned}$$

The first factor of this expression of the characteristic function of $\breve{X}_t$ tends towards 1 because $\lim _{t \uparrow 1/e} \sigma (t)=+\infty$ and the continuity of *f* on $\text{ cl }({\mathbb {D}}(0,1/e))$, while the second factor tends towards 1, as a consequence of (4.5). And thus, $\lim _{t\uparrow 1/e}\textbf{E}(e^{\imath \theta \breve{X}_t})=1$, for any $\theta \in {\mathbb {R}}$.

This means that $\breve{X}_t$ tends in distribution towards the constant 0, and not to the Gaussian distribution.

For the power series

$$\begin{aligned} f(z) = \exp \Big (\sum _{n = 1}^{\infty } \frac{n^{n-\delta }}{n!}\,z^n\Big ) \end{aligned}$$

and for the range $1/2< \delta < 3/2$, the same argument gives that $(\breve{X}_t)$ tends in distribution towards the constant 0, as $t \uparrow 1/e$.

Similarly, but using that for $3/2< \delta < 5/2$, we have that $\lim _{t \uparrow 1/e}m(t)$ is finite while $\lim _{t \uparrow 1/e}\sigma (t) = +\infty$, and also that for $\delta = 3/2$, we have that

$$\begin{aligned} m(t) \asymp \ln \Big (\frac{1}{1-et}\Big ) \quad \text{ and } \quad \sigma (t) \asymp \frac{1}{\sqrt{1-et}}, \quad \text { as } t \uparrow 1/e, \end{aligned}$$

we also obtain for the range $3/2 \le \delta < 5/2$, that the normalized Khinchin family $(\breve{X}_t)$ tends in distribution towards the constant 0, as $t \uparrow 1/e$.

Thus, the efg of the sets of functions ($\delta = 0$) is Gaussian, but the efgs of sets of trees ($\delta = 2$) and of sets of rooted trees ($\delta = 1$) are not Gaussian.

#### Question 1

Is

$$\begin{aligned} f(z)=\exp \Big (\sum _{n=1}^\infty \frac{n^{n-1/2}}{n!}\, z^n\Big ) \end{aligned}$$

Gaussian? It is not strongly Gaussian (see the definition below) since for its Khinchin family we have that

$$\begin{aligned} \lim _{t\uparrow 1/e} \frac{m(t)}{\sigma (t)}=(2\pi )^{-1/4}, \end{aligned}$$

while for strongly Gaussian power series it is always the case that this limit is $+\infty$. See formula (4.7) below.

### Strongly Gaussian Khinchin Families

The notion of strongly Gaussian power series *f* in ${\mathcal {K}}$ was introduced by Báez-Duarte in [2].

#### Definition 4.2

A power series $f \in {\mathcal {K}}$ and its Khinchin family $(X_t)_{t \in [0,R)}$ are termed *strongly Gaussian* if

$$\begin{aligned} \mathrm{a)} \quad \lim _{t \uparrow R} \sigma (t)=+\infty , \quad \text{ and }\quad \mathrm{b)} \quad \lim _{t \uparrow R} \int \nolimits _{|\theta | <\pi \sigma (t)}\big |\textbf{E}(e^{\imath \theta \breve{X}_t})-e^{-\theta ^2/2}\big | \, \mathrm{{d}}\theta =0. \end{aligned}$$

The exponential $f(z)=e^z$ is strongly Gaussian. In this case $\sigma (t)=\sqrt{t}$. This strong gaussianity follows from the gaussianity of $e^z$ and dominated convergence using the bound

$$\begin{aligned} \big |\textbf{E}(e^{\imath \theta \breve{X}_t})\big |=e^{t (\cos (\theta /\sqrt{t})-1)}\le e^{-2\theta ^2/\pi ^2}, \quad \text{ for }\ \theta \in {\mathbb {R}},\, t>0 \ \text{ such } \text{ that }\ |\theta |< \pi \sqrt{t}. \end{aligned}$$

As we will see in Theorem C, strongly Gaussian power series are Gaussian. Thus the power series $(1+z)^N$ and $1/(1-z)^N$, for $N \ge 1$, are all not strongly Gaussian.

For strongly Gaussian power series, we have the following key Theorem C. It appears in [11] for power series in the Hayman class. See Theorem A in [3] for a proof under the more general assumption of strongly Gaussian power series.

#### Theorem C

(Hayman’s central limit theorem) If the power series $f(z)=\sum _{n=0}^\infty a_n z^n$ in ${\mathcal {K}}$ is strongly Gaussian, then

4.6 $$\begin{aligned} \lim _{t \uparrow R} \sup \limits _{n \in {\mathbb {Z}}} \Big |\frac{a_n t^n}{f(t)} \sqrt{2\pi }\sigma (t) -e^{-(n-m(t))^2/(2\sigma ^2(t))}\Big |=0. \end{aligned}$$

Moreover,

$$\begin{aligned} \lim _{t \uparrow R}\textbf{P}( \breve{X}_t \le b)=\Phi (b), \quad \text{ for } \text{ every }\ b \in {\mathbb {R}}, \end{aligned}$$

and so, $(\breve{X}_t)$ converges in distribution towards the standard normal and *f* is Gaussian.

In this statement, $a_n=0$, for $n <0$.

By considering $n={-}1$ in (4.6), it follows that

4.7 $$\begin{aligned} \lim _{t \uparrow R}\dfrac{m(t)}{\sigma (t)}=+\infty , \end{aligned}$$

and, in particular, that $M_f=\infty$ for every $f\in {\mathcal {K}}$ that is strongly Gaussian.

The function $f(z)=e^{z^2}$ is Gaussian, because of Theorem A. Its variance function $\sigma ^2(t)=4t^2$ tends towards $\infty$ as $t \rightarrow \infty$, but *f* is not strongly Gaussian, since its Taylor coefficients of odd order are null and do not satisfy the asymptotic formula (4.8) below.

#### Coefficients of Strongly Gaussian Power Series

For strongly Gaussian power series, we have the following asymptotic formula for its coefficients.

##### Theorem D

(Hayman’s asymptotic formula) If $f(z)=\sum _{n=0}^\infty a_n z^n$ in ${\mathcal {K}}$ is strongly Gaussian, then

4.8 $$\begin{aligned} a_n \sim \frac{1}{\sqrt{2\pi }} \,\frac{f(t_n)}{t_n^n \,\sigma (t_n)}, \quad \text{ as }\ n \rightarrow \infty . \end{aligned}$$

In the asymptotic formula above, $t_n$ is given by $m(t_n)=n$, for each $n \ge 1$. This asymptotic formula follows readily from Theorem C, or alternatively, from Hayman’s formula (2.1) and strong gaussianity.

For the exponential function $f(z)=e^z$, one has $m(t)=t$ and $\sigma (t)=\sqrt{t}$, for $t \ge 0$, and $t_n=n$ for $n \ge 1$. The asymptotic formula above gives

$$\begin{aligned} \frac{1}{n!}\sim \frac{1}{\sqrt{2\pi }} \,\frac{e^n}{n^n \sqrt{n}}, \quad \text{ as }\ n \rightarrow \infty , \end{aligned}$$

that is Stirling’s formula.

Actually, if $\omega _n$ is a good approximation of $t_n$, in the sense that

$$\begin{aligned} \lim _{n \rightarrow \infty } \frac{m(\omega _n)-n}{\sigma (\omega _n)}=0, \end{aligned}$$

then from Hayman’s formula (2.1) and strong gaussianity we have that

4.9 $$\begin{aligned} a_n \sim \frac{1}{\sqrt{2\pi }}\,\frac{f(\omega _n)}{\sigma (\omega _n) \, \omega _n^n},\quad \text{ as }\ n \rightarrow \infty . \end{aligned}$$

In general, precise expressions for the $t_n$ are rare, since inverting *m*(*t*) is usually complicated. But, fortunately, in practice, one can do with a certain asymptotic approximation due to Báez-Duarte, [2], which we now describe.

Suppose that $f \in {\mathcal {K}}$ is strongly Gaussian. Assume that $\widetilde{m}(t)$ is continuous and monotonically increasing to $+\infty$ in [0, *R*) and that $\widetilde{m}(t)$ is a good approximation of *m*(*t*) in the sense that

4.10 $$\begin{aligned} \lim _{t \uparrow R} \frac{m(t)-\widetilde{m}(t)}{\sigma (t)}=0. \end{aligned}$$

Let $\tau _n$ be defined by $\widetilde{m}(\tau _n)=n$, for each $n \ge 1$.

##### Theorem E

(Báez-Duarte asymptotic formula) With the notations above, if $f(z)=\sum _{n=0}^\infty a_n z^n$ in ${\mathcal {K}}$ is strongly Gaussian and (4.10) is satisfied, then

$$\begin{aligned} a_n \sim \frac{1}{\sqrt{2\pi }} \,\frac{f(\tau _n)}{\tau _n^n \,\sigma (\tau _n)}, \quad \text{ as }\ n \rightarrow \infty . \end{aligned}$$

This follows readily from (4.9).

Besides, if $\widetilde{\sigma }(t)$ is such that $\sigma (t) \sim \widetilde{\sigma }(t)$ as $t \uparrow R$, we may further write

4.11 $$\begin{aligned} a_n \sim \frac{1}{\sqrt{2\pi }} \,\frac{f(\tau _n)}{\tau _n^n \,\widetilde{\sigma }(\tau _n)}, \quad \text{ as }\ n \rightarrow \infty . \end{aligned}$$

In practice, using (4.11) requires approximating *m* by $\widetilde{m}$ to obtain $\tau _n$, and then obtaining good enough approximations of $\sigma$ and *f* on (0, *R*) to produce asymptotic formulas of $\sigma (\tau _n)$ and $f(\tau _n)$.

### Hayman Class

The class of Hayman consists of power series *f* in ${\mathcal {K}}$ which satisfy some concrete and verifiable conditions which imply that *f* is strongly Gaussian, see Theorem F below.

#### Definition 4.3

A power series $f\in {\mathcal {K}}$ is in the *Hayman class* (or is Hayman-admissible or just *H*-admissible) if for a certain function $h:[0,R)\rightarrow (0, \pi ]$, the following conditions are satisfied:

4.12 $$\begin{aligned}{} & {} (\textrm{major}\ \textrm{arc}): \qquad \lim _{t \uparrow R} \sup _{|\theta |\le h(t)\, \sigma (t)} \big |\textbf{E}(e^{\imath \theta \breve{X}_t})\,e^{\theta ^2/2}-1\big |=0, \end{aligned}$$

4.13 $$\begin{aligned}{} & {} (\textrm{minor}\ \textrm{arc}): \qquad \lim _{t\uparrow R}\sigma (t) \, \sup _{h(t)\sigma (t)\le |\theta | \le \pi \sigma (t)} | \textbf{E}(e^{\imath \theta \breve{X}_t})|=0, \end{aligned}$$

and

4.14 $$\begin{aligned} (\textrm{variance}\ \textrm{condition}):\qquad \lim _{t \uparrow R} \sigma (t)=\infty . \end{aligned}$$

We refer to the function *h* in the definition above as a *cut* between a *major* arc and a *minor* arc.

Some authors include in the Hayman class power series with a finite number of negative coefficients. This is not the case in this paper.

For *f* in the Hayman class, the characteristic function of $\breve{X}_t$ is uniformly approximated by $e^{-\theta ^2/2}$ in the major arc, while it is uniformly $o(1/\sigma (t))$ in the minor arc.

Observe that condition (4.13) may be written more simply and in terms of *f* itself as the requirement that

$$\begin{aligned} \lim _{t\uparrow R}\sigma (t) \, \sup _{h(t)\le |\theta | \le \pi } | \textbf{E}(e^{\imath \theta X_t})| =\lim _{t\uparrow R}\sigma (t) \, \sup _{h(t)\le |\theta | \le \pi } \frac{|f(te^{\imath \theta })|}{f(t)}=0. \end{aligned}$$

#### Theorem F

Power series in the Hayman class are strongly Gaussian.

In a certain sense, the above theorem places the conditions for Hayman class as a criterion for being strongly Gaussian. For a proof, see Theorem 3.7 in [3]. Thus we have that being in the Hayman class implies strong gaussianity, which in turn implies gaussianity. In applications, we shall always check that the power series belongs to the Hayman class.

We refer to [3], and, of course, to [11], for examples and further properties of the Hayman class.

## Exponentials and the Hayman Class

Along this section, we consider nonconstant power series $g(z)=\sum _{n=0}^\infty b_n z^n$ with *nonnegative coefficients* and radius of convergence $R>0$; it is not required that $g(0)>0$. We are interested in conditions on *g* which guarantee that $f=e^g$, which is in ${\mathcal {K}}$, is in the Hayman class.

There are two results of Hayman in [11] along this line.

- If *g* is a power series in the Hayman class, then $f=e^g$ is in the Hayman class; see Theorem VI in [11].
- If *B* is a nonconstant polynomial with nonnegative coefficients and such that $Q_B=\gcd \{n \ge 1: b_n >0\}=1$, then $e^B$ is in the Hayman class.

### Remark 5.1

In [13], it is shown that if *g* is in the Hayman class, then $f=e^g$ satisfies the stronger (than Hayman) conditions of Harris and Schoenfeld, [10], which allow to obtain full asymptotic expansions of coefficients and not just asymptotic formulas.

### Remark 5.2

In Theorem X of [11], Hayman proves the following stronger version of (b): *Let* $B(z)=\sum _{n=0}^N b_n z^n$ *be a nonconstant polynomial with real coefficients such that for each* $d>1$ *there exists* *m*, *not a multiple of* *d*, *such that* $b_m \ne 0$, *and such that if* *m*(*d*) *is the largest such* *m*, *then* $b_{m(d)}>0$. *If* $e^B$ *is in* ${\mathcal {K}}$, *then* $e^B$ *is in the Hayman class*.

For a direct proof of E2), we refer to Proposition 5.1 in [3].

Theorem G, stated below, which is Theorem 4.1 in [3], exhibits conditions on the function *g* which ensure that *f* is in the Hayman class.

Further, we describe in Theorem 5.3 a ‘practical’  approach to verify that a power series satisfies the conditions of Theorem G.

Finally, Theorems 5.5 and 5.7 give easily verifiable conditions on *the coefficients* $b_n$ *of* *g* that imply that $f=e^g$ is in the Hayman class. These theorems may be directly applied to the generating functions of sets constructions (and also to other exponentials) to check that they belong to the Hayman class.

Let $(X_t)_{t \in [0,R)}$ be the Khinchin family of $f=e^g$. The mean and variance functions of *f*, *written in terms of* *g*, are

5.1 $$\begin{aligned} m(t)=\frac{tf'(t)}{f(t)} = t g^\prime (t)=\sum _{n=1}^\infty n b_n \,t^n, \quad \text{ for }\ t \in (0,R), \end{aligned}$$

and

5.2 $$\begin{aligned} \sigma ^2(t)=tm'(t)=tg^\prime (t)+t^2g^{\prime \prime }(t) =\sum _{n=1}^\infty n^2 b_n \,t^n,\quad \text{ for }\ t \in (0,R). \end{aligned}$$

Since *g* has nonnegative coefficients, the variance function $\sigma ^2(t)$ of *f* is increasing in [0, *R*). Observe also that $m(t)\le \sigma ^2(t)$, for $t \in [0,R)$.

The variance condition (4.14) required for *f* to be in the Hayman class translates readily in the following condition in terms of *g*:

$$\begin{aligned} \lim _{t \uparrow R} \big (tg^\prime (t) +t^2g^{\prime \prime }(t)\big )=+\infty . \end{aligned}$$

To properly handle the minor and major arc conditions in terms of *g*, we introduce

5.3 $$\begin{aligned} \omega _g(t)\triangleq \frac{1}{6}(b_1 t+8 b_2 t^2 +\frac{9}{2} \,t^3g^{\prime \prime \prime }(t)) , \quad \text{ for }\ t \in (0,R). \end{aligned}$$

The following theorem is Theorem 4.1 in [3].

### Theorem G

Let *g* be a nonconstant power series with radius of convergence $R>0$ and nonnegative coefficients.

If the variance condition

5.4 $$\begin{aligned} \lim _{t \uparrow R} \big (tg^\prime (t) +t^2g^{\prime \prime } (t)\big )=+\infty \end{aligned}$$

is satisfied and there is a cut function $h:[0,R) \rightarrow (0,\pi )$ so that

5.5 $$\begin{aligned} \lim _{t \uparrow R} \omega _g(t)\, h(t)^3=0, \end{aligned}$$

and

5.6 $$\begin{aligned} \lim _{t \uparrow R} \sigma (t) \exp \Big (\sup \limits _{h(t)\le |\theta |\le \pi } \Re g(te^{\imath \theta })-g(t)\Big )=0 \end{aligned}$$

hold, then $f=e^g$ is in the Hayman class.

Condition (5.5) of Theorem G gives that the cut function *h* fulfills condition (4.12) on the major arc. Also, condition (5.6) of Theorem G, which involves *h* and *g*, implies that condition (4.13) on the minor arc is satisfied.

Condition (5.6) on the minor arc is the most delicate to check. In practice, it depends on properly bounding $\sup \nolimits _{\omega \le |\theta |\le \pi }\Re g(te^{\imath \theta })-g(t)$ for general $\omega$, as it is exhibited in the following variant, actually a corollary, of Theorem G.

### Theorem 5.3

Let *g* be a nonconstant power series with radius of convergence *R* and nonnegative coefficients, and let $h:[0,R) \rightarrow (0,\pi )$ be a cut function.

Assume that the variance condition (5.4) and the condition on the major arc (5.5) of Theorem G are satisfied.

Assume further that there are positive functions *U*, *V* defined in $(t_0,R)$, for some $t_0\in (0,R)$, where *U* takes values in $(0,\pi ]$ and *V* in $(0,\infty )$, and such that

5.7 $$\begin{aligned} \sup \limits _{|\theta |\ge \omega }\big (\Re g(t e^{\imath \theta })-g(t)\big )\le -V(t) \, \omega ^2, \quad \text{ for } \ \omega \le U(t)\ \text{ and }\ t \in (t_0,R), \end{aligned}$$

and

5.8 $$\begin{aligned} h(t)\le U(t), \quad \text{ for }\ t \in (t_0,R) \quad \text{ and } \quad \lim _{t \uparrow R} \sigma (t) \, e^{-V(t) h(t)^2}=0. \end{aligned}$$

Then $f=e^g$ is in the Hayman class.

### Proof

The pair of conditions (5.7) and (5.8) together imply condition (5.6). This is so since the first half of (5.8) allows us to take $\omega =h(t)$ in (5.7) to obtain

$$\begin{aligned} \exp \left( \sup \limits _{|\theta |\ge h(t)}\Re g(t e^{\imath \theta }) -g(t)\right) \le \exp \big (-V(t) \, h(t)^2\big ), \quad \text{ for }\ t\in (t_0,R), \end{aligned}$$

and to then apply the second half of (5.8). $\square$

Notice further that if for a function *V* defined in $(t_0,R)$, for some $t_0\in (0,R)$, and taking values in $(0,+\infty )$, we have that

5.9 $$\begin{aligned} \Re g(t e^{\imath \theta })-g(t)\le -V(t) \, \theta ^2, \quad \text{ for }\ |\theta |\le \pi \ \text{ and }\ t \in (t_0,R), \end{aligned}$$

then we may set $U\equiv \pi$, and the cut *h* is just required to satisfy

5.10 $$\begin{aligned} \lim _{t \uparrow R} \sigma (t) \, e^{-V(t) h(t)^2}=0. \end{aligned}$$

Thus these two conditions (5.9) and (5.10) imply the conditions (5.7) and (5.8) of Theorem 5.3.

Next we will exhibit easily verifiable requirements on the coefficients of *g* to obtain functions *V* and *U* so that the conditions (5.7) and (5.8) hold, or, simply, a function *V* so that the conditions (5.9) and (5.10) are satisfied.

The announced requirements differ if the power series *g* is entire or has finite radius of convergence; we split the discussion accordingly.

### Case *g* Entire, $R=\infty$

Here, *g* is an entire power series with nonnegative coefficients.

#### Lemma 5.4

If an entire power series *g* with nonnegative coefficients satisfies, for some $\beta >0$ and $B>0$, that

5.11 $$\begin{aligned} \Re g(te^{\imath \theta })-g(t)\le B(e^{\beta t \cos \theta } -e^{\beta t}), \quad \text{ for }\ t >0\ \text{ and }\ |\theta |\le \pi , \end{aligned}$$

then condition (5.9) is satisfied with $V(t)=C e^{\beta t}$ for some constant $C>0$ depending only on *B* and $\beta$, and condition (5.10) requires that $\lim _{t \rightarrow \infty } \sigma (t) \exp (- C e^{\beta t} h(t)^2)=0$.

#### Proof

For $t>0$ and $\beta >0$, the convexity of the exponential function $x \mapsto e^{\beta t x}$ in the interval $[-1,1]$ gives

$$\begin{aligned} e^{\beta t}-e^{\beta t \cos \theta }\ge (1-\cos \theta ) \,\frac{e^{\beta t}-e^{-\beta t}}{2}, \quad \text {for}\ \theta \in [-\pi ,\pi ]. \end{aligned}$$

Thus, we have, for $t\ge 1$, that

$$\begin{aligned} e^{\beta t \cos \theta }-e^{\beta t}\le & {} (\cos \theta -1)\, \frac{e^{\beta t}-e^{-\beta t}}{2} \le e^{\beta t}\, \frac{1-e^{-2 \beta }}{2}\, (\cos \theta -1)\\\le & {} -\frac{1-e^{-2 \beta }}{\pi ^2}\, e^{\beta t} \,\theta ^2. \end{aligned}$$

In the second inequality, we have used that $t\ge 1$; the inequality $1-\cos x\ge 2x^2/\pi ^2$, valid for $x\in [-\pi ,\pi ]$, yields the last step. $\square$

#### Theorem 5.5

Let $$g(z)=\sum _{n=0}^{\infty } b_n z^n$$ satisfy

5.12 $$\begin{aligned} B \,\frac{\beta ^n}{n!} \le b_n\le L\,\frac{\lambda ^n}{n!} \quad \text{ for }\ n \ge 1, \end{aligned}$$

for some constants $B, L>0$, and $\beta , \lambda \ge 0$ such that $2\lambda <3\beta$.

Then *g* satisfies the requirements of Theorem 5.3 and, therefore, $f=e^g$ is in the Hayman class.

#### Proof

Condition (5.12) gives that the power series *g* has radius of convergence $R=+\infty$. Besides, condition (5.12) and Proposition 2.3 show that the variance $\sigma ^2(t)$ satisfies that $t^2 e^{\beta t}=O(\sigma ^2(t))$ and that $\sigma ^2(t)=O( t^2 e^{\lambda t})$, and that $\omega _g$ satisfies that $\omega _g(t)=O(t^3 e^{\lambda t})$, as $t \rightarrow \infty$.

The variance condition (5.4) is thus obviously satisfied. The major arc condition (5.5) holds with the cut function $h(t)=e^{-\alpha t}$, where $\alpha \in (\lambda /3,\beta /2)$, since $3\alpha >\lambda$.

Also, for $t >0$ and $|\theta |\le \pi$, we have that

$$\begin{aligned} \Re g(te^{\imath \theta })-g(t)= & {} \sum _{n=1}^\infty b_n \, t^n (\cos n \theta -1)\le B \sum _{n=1}^\infty \frac{(\beta t)^n}{n!} (\cos n \theta -1)\\= & {} B\big (\Re e^{\beta t e^{\imath \theta }} -e^{\beta t}\big )\le B ( |e^{\beta t e^{\imath \theta }} | -e^{\beta t} )=B (e^{\beta t \cos \theta }-e^{\beta t} ), \end{aligned}$$

and, consequently, condition (5.11) of Lemma 5.4 is satisfied.

Condition (5.9) is satisfied with the choice $V(t)=Ce^{\beta t}$, $t>1$, and (5.10) holds because $2\alpha <\beta$. As mentioned above, these two conditions imply the conditions (5.7) and (5.8) of Theorem 5.3, and thus we conclude that *f* is in the Hayman class. $\square$

### Case *g* Not Entire, $R<\infty$

Here, we will repeatedly appeal to Proposition 2.1.

#### Lemma 5.6

If a power series *g* with nonnegative coefficients and radius of convergence $R=1$ satisfies, for some $\beta >0$ and $B>0$, that

5.13 $$\begin{aligned} \Re g(t e^{\imath \theta })-g(t)\le B \Big (\frac{1}{|1-te^{\imath \theta }|^\beta } -\frac{1}{(1-t)^\beta }\Big ), \quad \text{ for }\ t\in (0,1) \ \text{ and }\ |\theta |\le \pi , \end{aligned}$$

then

$$\begin{aligned} \sup \limits _{|\theta |\ge \omega } \Re g(te^{\imath \theta })-g(t)\le & {} - C \, \frac{1}{(1-t)^{2+\beta }}\, \omega ^2,\\{} & {} \quad \text{ for }\ t \in (1/2, 1)\ \text{ and }\ 0\le \omega \le D (1-t), \end{aligned}$$

where $C>0$ and $D>0$ depend only on $\beta$ and *B*.

And, in particular, if we set $V(t)= C/(1-t)^{2+\beta }$, and $U(t)=D(1-t)$, for $t\in (1/2,1)$, then condition (5.7) is satisfied, and condition (5.8) requires that

$$\begin{aligned} \lim _{t \uparrow 1} \sigma (t) \exp \big (- C h(t)^2/(1-t)^{2+\beta }\big )=0. \end{aligned}$$

#### Proof

Let $\beta >0$. For $t \in (0,1)$ and $|\theta |\le \pi$, we have that

$$\begin{aligned} \Re g(t e^{\imath \theta })-g(t)\le \frac{B}{(1-t)^\beta } \Big (\Big (\Big |\frac{1-t}{1-te^{\imath \theta }}\Big |^2 \Big )^{\beta /2}-1\Big ). \end{aligned}$$

Thus, for $t\in (1/2, 1)$ and $0\le \omega <(1-t)$ we have that

$$\begin{aligned} \sup \limits _{|\theta |\ge \omega } \Re g(t e^{\imath \theta }) -g(t)\le \frac{B}{(1-t)^\beta } \Big (\big (1-C \frac{1}{(1-t)^2}\, \omega ^2\big )^{\beta /2}-1\Big ), \end{aligned}$$

and, for $\omega <D_\beta (1-t)$, for $D_\beta >0$ appropriately small, and some constant $C_\beta >0$, we have that

$$\begin{aligned} \sup \limits _{|\theta |\ge \omega } \Re g(t e^{\imath \theta }) -g(t)\le -\frac{B}{(1-t)^\beta }\, C_\beta \,\frac{1}{(1-t)^2}\, \omega ^2. \end{aligned}$$

$$\square $$

#### Theorem 5.7

Let $$g(z)=\sum _{n=0}^{\infty } b_n z^n$$ satisfy

5.14 $$\begin{aligned} B \,\frac{n^\beta }{R^n} \le b_n\le L \,\frac{n^\lambda }{R^n} , \quad \text{ for }\ n \ge 1, \end{aligned}$$

for some constants $B, L>0$, finite radius $R>0$ and $\beta , \lambda >-1$ such that $2\lambda <3\beta +1$.

Then *g* satisfies the requirements of Theorem 5.3 and, therefore, $f=e^g$ is in the Hayman class.

#### Proof

Because of (5.14), the power series *g* has radius of convergence *R*. By considering *g*(*Rz*), we may assume that $R=1$. Appealing to Proposition 2.1, we see that the variance function of $f=e^g$ satisfies that ${1}/{(1-t)^{3+\beta }}=O(\sigma ^2(t))$ and that $\sigma ^2(t)=O( {1}/{(1-t)^{3+\lambda }})$, and that $\omega _g$ satisfies that $\omega _g(t)=O( {1}/{(1-t)^{4+\lambda }} )$ as $t \uparrow 1$.

The variance condition (5.4) (or directly (4.14)) is obviously satisfied.

We propose a cut $h(t)=(1-t)^{\alpha }$, where $\alpha \in (\lambda /3+4/3, \beta /2+3/2)$. The major arc condition (5.5) holds since $3\alpha >\lambda +4$.

For $t \in (0,1)$ and $|\theta |\le \pi$, we have that

$$\begin{aligned} \Re g(t e^{\imath \theta })-g(t)= & {} \sum _{n=1}^{\infty } b_n \,t^n (\cos n\theta -1)\le B\sum _{n=1}^{\infty } n^\beta \, t^n (\cos n\theta -1)\\\le & {} B_\beta \sum _{n=1}^{\infty } \frac{\Gamma (n+\beta +1)}{\Gamma (\beta +1) n!}\, t^n (\cos n\theta -1)\\= & {} B_\beta \Big (\Re \frac{1}{(1-te^{\imath \theta })^{\beta +1}} -\frac{1}{(1-t)^{\beta +1}}\Big )\\\le & {} B_\beta \Big (\Big |\frac{1}{1-te^{\imath \theta }} \Big |^{\beta +1}-\Big (\frac{1}{1-t}\Big )^{\beta +1}\Big ), \end{aligned}$$

where we have appealed to the comparison (2.2) within Proposition 2.1. Consequently, condition (5.13) of Lemma 5.6 does hold.

If we set $V(t)= C/(1-t)^{\beta +3}$, for $t \in (1/2,1)$, (5.7) is satisfied and (5.8) requires that $\lim _{t \uparrow 1} \sigma (t) \exp (- C h(t)^2/(1-t)^{\beta +3})=0$, which does hold since $2 \alpha <\beta +3$.

Thus, the conclusion follows from Theorem 5.3. $\square$

### Applications of Theorems 5.5 and 5.7

#### Sets of Labeled Classes

$\bullet$ The egf $e^{e^z-1}$ of the Bell numbers, enumerating *sets of sets*, is $f=e^g$ with $b_n=1/n!$, for $n \ge 1$.

The egf $e^{ze^z}$ of *sets of pointed sets*, is $f=e^g$ with $b_n=n/n!$, for $n \ge 1$.

Both satisfy the hypothesis of Theorem 5.5 and, thus, in particular they are in the Hayman class.

$\bullet$ The power series $f(z)=e^{z/(1-z)}$, egf of sets of lists, has $g(z)=z/(1-z)$ with $b_n=1$, for $n \ge 1$. The coefficients of the function *g* satisfy the hypothesis of Theorem 5.7 and, thus, in particular, $f=e^g$ is in the Hayman class.

In general, $f(z)=e^{z/(1-z)^\gamma }$ is in the Hayman class for $\gamma >0$.

But $f(z)=e^{-\ln (1-z)}=1/(1-z)$, the egf of *sets of cycles* is not even Gaussian, as shown in Sect. 4.1.

$\bullet$ The egf *f* of *sets of functions* is $f=e^g$ with $g(z)=\sum _{n=1}^\infty (n^n/n!) z^n$. In this case, the coefficients $b_n$ of *g* satisfy (5.14) with $R=1/e$ and $\beta =\lambda =-1/2$, and therefore *f* is in the Hayman class.

In general, the exponential of $\sum _{n=1}^\infty (n^{n{-}\alpha }/n!) \,z^n$ is in the Hayman class if $0\le \alpha <1/2$. As we have seen in Sect. 4.1, for $\alpha =1/2$ it is not strongly Gaussian, and for $\alpha >1/2$ is not even Gaussian.

#### Sets of Unlabeled Classes

$\bullet$ For the ogf *P* of partitions, we have that $P=e^g$, with

$$\begin{aligned} g(z)=\sum _{n=1}^\infty \frac{\sigma _1(n)}{n}\, z^n, \end{aligned}$$

where $\sigma _1(n)$ denotes the sum of divisors of the positive integer *n*. The coefficients $b_n=\sigma _1(n)/n$ satisfy, in this case,

$$\begin{aligned} 1\le b_n \le D_\varepsilon n^\varepsilon , \end{aligned}$$

for each $\varepsilon >0$ and some constant $D_\varepsilon >0$. See Theorem 322 in [9]. (Actually, we may bound from above with $\ln \ln n$, see Theorem 323 in [9]). Therefore the function *g* satisfies the hypothesis of Theorem 5.7 and this means, in particular, that $P=e^g$ is in the Hayman class.

The same argument gives that the infinite product $\prod _{j=1}^\infty 1/(1-z^j)^{c_j}$, where the $c_j$ are integers satisfying $1\le c_j\le c$, for some constant $c>1$, is in the Hayman class.

$\bullet$ For the ogf *Q* of partitions into distinct parts, given by

$$\begin{aligned} Q(z){\mathop {=}\limits ^{(1)}}\prod _{j=1}^\infty (1+z^j), \quad \text{ or } \text{ alternatively, } \text{ by } \quad Q(z) {\mathop {=}\limits ^{(2)}}\prod _{j=0}^\infty \frac{1}{1-z^{2j+1}}, \end{aligned}$$

we have that $Q=e^g$, where

$$\begin{aligned} g(z){\mathop {=}\limits ^{\textrm{by}\ (1)}}\sum _{k,j \ge 1}\frac{(-1)^{k+1}}{k} \,z^{kj}{\mathop {=}\limits ^{\textrm{by} \ (2)}}\sum _{k\ge 1; j\ge 0}\frac{1}{k} \,z^{k(2j+1)} =\sum _{n=1}^\infty \frac{\sigma ^{\textrm{odd}}_1(n)}{n} \, z^n, \end{aligned}$$

where $\sigma ^{\textrm{odd}}_1(n)$ registers the sum of the odd divisors of *n*.

The coefficients $b_n=\sigma ^{\textrm{odd}}_1(n)/n$ satisfy

5.15 $$\begin{aligned} \frac{1}{n}\le b_n \le D_\varepsilon n^\varepsilon , \end{aligned}$$

for each $\varepsilon >0$ and some constant $D_\varepsilon >0$. The inequality on the left holds simply because $1\mid n$, while the inequality on the right holds because $\sigma ^{\textrm{odd}}_1(n)\le \sigma _1(n)$ and Theorem 322 in [9].

These bounds, though, are not within reach of Theorem 5.7, and for the function *Q* we will verify directly the hypothesis of Theorem 5.3.

Using (5.15), Proposition 2.1 and formula (5.2), we have for the variance function $\sigma ^2(t)$ of $f=e^g$ that

$$\begin{aligned} \dfrac{1}{(1-t)^{2}}=O(\sigma ^2(t)) \quad \text{ and } \quad \sigma ^2(t)=O\Big (\dfrac{1}{(1-t)^{3+\varepsilon }}\Big ), \quad \text{ as }\ t \uparrow 1, \end{aligned}$$

and for the function $\omega _g(t)$ that $\omega _g(t)=O({1}/{(1-t)^{4+\varepsilon }})$, as $t \uparrow 1$.

The variance condition (5.4) (or directly (4.14)) is obviously satisfied. We propose a cut $h(t)=(1-t)^\alpha$, where $\alpha \in (4/3, 3/2)$.

The major arc condition (5.5) holds since $3\alpha >4+\varepsilon$, for appropriate small $\varepsilon$.

For $t \in (0,1)$ and $|\theta |\le \pi$, and $z=te^{\imath \theta }$, we have that

$$\begin{aligned} \Big |\frac{1+te^{\imath \theta }}{1+t}\Big |^2=1 +\frac{2t (\cos \theta -1)}{(1+t)^2}\le 1+\frac{t}{2} (\cos \theta -1)\le e^{(t/2 (\cos \theta -1)}=e^{(\Re z -|z|)/2}, \end{aligned}$$

and, so,

$$\begin{aligned} \frac{|Q(z)|}{Q(|z|)}\le \exp \Big ( \frac{1}{4} \Big (\Re \frac{z}{1-z} -\frac{|z|}{1-|z|}\Big )\Big ) = \exp \Big ( \frac{1}{4} \Big (\Re \frac{1}{1-z} -\frac{1}{1-|z|}\Big )\Big ), \end{aligned}$$

and, consequently,

$$\begin{aligned} \Re g(z) -g(|z|)=\ln \frac{|Q(z)|}{Q(|z|)} \le \frac{1}{4} \Big (\Re \frac{1}{1-z} -\frac{1}{1-|z|}\Big ) \le \Big |\frac{1}{1-z}\Big | -\frac{1}{1-|z|}\cdot \end{aligned}$$

Lemma 5.6 gives us $V(t)=C/(1-t)^3$ and $U(t)=D(1-t)$. Now, condition (5.8) is satisfied since $\alpha <3/2$.

Thus, the function *g* satisfies the conditions of Theorem 5.3, and, in particular, $Q=e^g$ is in the Hayman class.

$\bullet$ For the ogf *M* of plane partitions (see [6], p. 580),

$$\begin{aligned} M(z)=\prod _{j=1}^\infty \frac{1}{(1-z^j)^j}, \end{aligned}$$

we have that $M=e^g$, with *g* given by

$$\begin{aligned} g(z)=\sum _{n=1}^\infty \frac{\sigma _2(n)}{n} \,z^n, \end{aligned}$$

where $\sigma _2(n)$ denotes the sum of the squares of the divisors of the integer $n \ge 1$. For each $\varepsilon >0$, there is a constant $C_\varepsilon >0$ such that

$$\begin{aligned} n\le \frac{\sigma _2(n)}{n}\le C_\varepsilon \, n^{1+\varepsilon }, \quad \text{ for } \text{ each }\ n \ge 1. \end{aligned}$$

This follows since $\sigma _2(n)\le n \sigma _1(n)$ and $\sigma _1(n)\le C_\varepsilon n^{1+\varepsilon }$. Thus we see that *g* satisfies the conditions of Theorem 5.7, and, in particular, we obtain that $M=e^g$ is in the Hayman class.

Likewise, and more generally, we see that for integer $c\ge 0$, the ogf of colored partitions,

$$\begin{aligned} \prod _{j=1}^\infty \frac{1}{(1-z^j)^{j^c}}, \end{aligned}$$

where each part *j* appears in $j^c$ different colors, is in the Hayman class. Observe that $n^c\le \sigma _{c+1}(n)/n\le C_\varepsilon \,n^{c+\varepsilon }$, for $n \ge 1$.

##### Question 2

For the number $p_s(n)$ of partitions into squares (i.e., partitions whose parts are whole squares) we have that

$$\begin{aligned} S(z)\triangleq \prod _{j=1}^\infty \frac{1}{1-z^{j^2}} =\sum _{n=0}^\infty p_s(n) \,z^n. \end{aligned}$$

Is *S* Gaussian or strongly Gaussian? See [7, 15]. Theorem 5.7 is not applicable: the corresponding *g* is

$$\begin{aligned} g(z)=\sum _{m=1}^\infty \frac{1}{m} \Big (\sum _{j^2\mid m} j^2\Big ) \,z^m, \end{aligned}$$

and $\sum _{j^2\mid m} j^2=1$ for any *m* which is a product of distinct primes.

Same question for

$$\begin{aligned} \prod _{j=1}^\infty \frac{1}{1-z^{2^j}}, \end{aligned}$$

whose coefficients count the number of partitions whose parts are powers of 2. See [4]. Theorem 5.7 is not applicable, either.

##### Question 3

The infinite product $\prod _{j=1}^\infty (1+z^j)^{j^c}$ has nonnegative coefficients. Is it Hayman?

## On Asymptotic Formulas of Coefficients

Once you know that a power series *f* is in the Hayman class, and thus that *f* is strongly Gaussian, you may use the asymptotic formula of Hayman of Theorem D, or even better, the asymptotic formula of Báez-Duarte of Theorem E, to yield an asymptotic formula for the coefficients of *f*.

As a token of the general approach, consider the partition function *P*. Hayman’s formula gives us that

6.1 $$\begin{aligned} p(n)\sim \frac{1}{\sqrt{2\pi }} \,\frac{P(t_n)}{t_n^n\, \sigma (t_n)}, \quad \text{ as }\ n \rightarrow \infty , \end{aligned}$$

where *m*(*t*) and $\sigma ^2(t)$ are the mean and variance functions of *P* and $t_n$ is such that $m(t_n)=n$.

As such, this formula (6.1) is too implicit, of course, on three counts: $t_n$ is not explicit, and hardly ever is, and, besides, the formula involves $\sigma (t_n)$ and $P(t_n)$.

We proceed by appealing to the Báez-Duarte formula (4.11), and by obtaining, on the one hand, asymptotic formulas for $\tau _n$ and $\widetilde{\sigma }(\tau _n)$, and for $P(\tau _n)$ on the other hand, as follows (see the details in Section 6.3.1 of [3]).

*Concerning* $\tau _n$ *and* $\sigma (\tau _n)$. By means of Euler’s summation, we may approximate the mean function *m*(*t*) by

$$\begin{aligned} m(e^{-s})\sim \frac{\zeta (2)}{s^2}\triangleq \widetilde{m}(e^{-s}), \quad \text{ as }\ s\downarrow 0, \end{aligned}$$

and $$\sigma ^2(t)$$ by

$$\begin{aligned} \sigma ^2(e^{-s})\sim \frac{2\zeta (2)}{s^3} \triangleq \widetilde{\sigma }^2(e^{-s}), \qquad \text{ as }\ s \downarrow 0, \end{aligned}$$

while checking that condition (4.10) is satisfied.

Now if $\tau _n$ is given by $\tau _n=e^{-s_n}$ and $s_n=\sqrt{\zeta (2)/n}$ so that $\widetilde{m}(\tau _n)= n$, we may appeal to the formula of Báez-Duarte (4.11) and write

6.2 $$\begin{aligned} p(n)\sim \frac{1}{\sqrt{2\pi }}\,\frac{P(\tau _n)}{\tau _n^n \,\widetilde{\sigma }(\tau _n)}, \quad \text{ as }\ n \rightarrow \infty . \end{aligned}$$

*Concerning* $P(\tau _n)$. Finally, Euler’s summation again gives that

$$\begin{aligned} P(e^{-s})\sim \frac{1}{\sqrt{2\pi }} \,\sqrt{s} \,e^{\zeta (2)/s}, \quad \text{ as }\ s \downarrow 0, \end{aligned}$$

and thus

$$\begin{aligned} P(e^{-s_n})\sim \frac{1}{\sqrt{2\pi }} \,\sqrt{s_n} \,e^{\zeta (2)/s_n}, \quad \text{ as }\ n \rightarrow \infty . \end{aligned}$$

Upon substitution in (6.2), the Hardy–Ramanujan asymptotic formula for partitions follows:

$$\begin{aligned} p(n)\sim \frac{1}{4\sqrt{3}} \,\frac{1}{n} \, e^{2\sqrt{\zeta (2)}\sqrt{n}},\quad \text{ as }\ n\rightarrow \infty . \end{aligned}$$

In short, this is the basic procedure for obtaining asymptotic formulas for coefficients of power series in the Hayman class. For a number of detailed examples, including the partition function above, general set constructions, and a few others, we refer the reader again to [3].
